# Machine Learning-Empowered Electromagnetic Wave Absorbing Materials: From Forward Prediction to Generative Inverse Design

**DOI:** 10.3390/molecules31142408

**Published:** 2026-07-08

**Authors:** Tongbaihui Qi, Jintang Zhou

**Affiliations:** 1 Shanghai Aircraft Design & Research Institute, Commercial Aircraft Corporation of China, Ltd. (COMAC), Shanghai 201210, China; 2 College of Materials Science and Technology, Nanjing University of Aeronautics and Astronautics, Nanjing 210016, China; imzjt@126.com

**Keywords:** electromagnetic wave absorbing materials, machine learning, inverse design, generative models, physics-informed learning, AI agents

## Abstract

Electromagnetic wave absorbing materials are important for electromagnetic protection, radar stealth, wireless communication, and advanced electronic systems. However, traditional design methods mainly rely on repeated experiments and full-wave simulations, which are time-consuming and inefficient when dealing with complex compositions, microstructures, and multilayer structures. Machine learning provides a new route to accelerate the design of high-performance absorbers by learning the relationship among material composition, structure, electromagnetic parameters, and absorption performance. This review summarizes recent progress in machine-learning-empowered electromagnetic wave absorbing materials. First, the basic physical principles of electromagnetic wave absorption are introduced, including reflection loss, impedance matching, attenuation, and physical limits such as the Rozanov and Snoek limits. Then, typical machine learning models are discussed, including classical machine learning, deep learning, generative models, physics-informed models, large language models, and artificial-intelligence (AI) Agents. Their applications are further summarized from forward property prediction, high-throughput screening, inverse design, electromagnetic parameter decoupling, physics-informed modeling, explainability, multi-objective optimization, and data augmentation. Finally, the main challenges and future directions are discussed, including data standardization, physics-guided learning, foundation models, autonomous laboratories, and engineering-scale validation. This review shows that machine learning is changing absorber research from experience-driven trial-and-error to data-driven and knowledge-driven design, and provides a useful reference for developing next-generation electromagnetic wave absorbing materials.

## 1. Introduction

### 1.1. The Importance of Electromagnetic Wave Absorbing Materials

The fast growth of 5G/6G wireless, millimeter-wave radar, autonomous driving sensors, satellite internet, and high-density IoT devices has turned the electromagnetic spectrum into a strategic resource on par with land and energy. Electromagnetic interference (EMI) and electromagnetic pollution now threaten the reliability of precision electronics, information security, and human health [1,2]. Electromagnetic wave absorbing materials dissipate electromagnetic energy and suppress unwanted reflections, and serve as key enabling materials for next-generation information technology, aerospace, smart equipment, and green electronics. Their performance sets the upper limit of high-end electronic devices and electromagnetic systems [3,4,5]. An ideal absorber should stay thin and light, cover a wide band, and absorb strongly at the same time, yet these goals conflict by nature: a thin layer is hard to keep wide-band, and strong absorption often comes from narrow-band resonance [2]. Designing high-performance absorbers thus reduces to a multi-objective problem bounded by physical limits, and the field needs a new methodology to push further.

### 1.2. Limitations of the Traditional Research Paradigm

**Figure 1:**
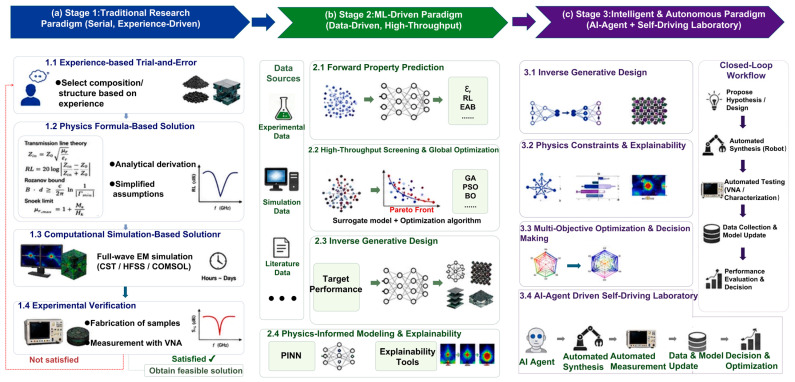
Paradigm Shift in Absorber Material Design: From Traditional Trial-and-Error Research to Machine-Learning-Driven and Autonomous Closed-Loop Discovery.

For a long time, research on absorbing materials has followed a serial paradigm of trial-and-error experiments plus full-wave electromagnetic simulations. As shown in Figure 1, Stage 1, researchers pick a composition or structure from experience, fabricate samples, measure the electromagnetic parameters with a vector network analyzer (VNA), and then adjust the recipe, or they sweep parameters in commercial simulators such as CST, HFSS, or COMSOL with Python 3.12 [6]. The design space of absorbing materials is huge, since it couples chemical composition, crystal phase, hetero-interfaces, micro-morphology, and macro-topology into a nearly infinite parameter space [7]. A single experimental loop takes days to weeks, and a single high-fidelity full-wave simulation can take hours, so orthogonal experiments and grid sweeps quickly become impractical in both coverage and efficiency. Experience-driven local search rarely produces breakthrough topologies, and struggles to approach theoretical limits such as the Rozanov thickness–bandwidth bound [8] or the Snoek permeability bound [9]. The bottleneck of absorbing-material research today is no longer the material systems but the research methodology, where data-driven approaches step in.

### 1.3. The Paradigm Shift Brought by Machine Learning

Machine learning (ML) and artificial intelligence (AI) provide a systematic solution to this methodological bottleneck [10,11,12,13,14,15,16,17,18,19,20,21,22,23,24,25,26,27]. As shown in Figure 1, Stage 2, by learning the high-dimensional mapping among composition, structure, and property directly from experimental or simulation data, AI serves as a surrogate that replaces expensive evaluations, and now covers the whole research pipeline [28,29,30,31,32,33,34,35,36,37,38,39,40]. The pipeline includes forward property prediction, where random forest (RF), extreme gradient boosting (XGBoost), and deep neural networks (DNN) learn $\varepsilon_{r},{\;\mu }_{r}$, the reflection loss (RL), and the effective absorption bandwidth (EAB) [41]; high-throughput screening and global optimization, where surrogate models couple with genetic algorithms (GA), particle swarm optimization (PSO), or Bayesian optimization (BO) [42]; inverse generative design, where variational autoencoders (VAE), generative adversarial networks (GAN), and mixture density networks (MDN) map a target performance back to a material structure and handle the one-to-many mapping problem of inverse design [43]; physics-informed modeling and explainability, where physics-informed neural networks (PINN), SHapley Additive exPlanations (SHAP) analysis, and Eigen-CAM expose the physical mechanisms behind the predictions [44]; and AI-Agent-driven self-driving laboratories that run the closed loop of hypothesis, experiment, test, and update on their own [5], as shown in Figure 1, Stage 3. The shift from experience-driven research to data- and knowledge-driven research lets tasks that used to take months or years finish in weeks, and opens a path to systematically approach the performance ceilings set by physics [45,46,47,48,49,50,51,52].

### 1.4. Existing Reviews: Contributions and Gaps

Several recent reviews have summarized absorbing materials from different angles. Durairaj and Murugesan (2025) reviewed EMI shielding materials, covering conductive polymers, graphene-based composites, MXenes, and metamaterials, with machine learning mentioned only as a future outlook [3]. Guo et al. (2025) published a critical review on Sub-8 GHz low-frequency absorbers, tracing the evolution from traditional magnetic materials to next-generation multifunctional composites, and listing ML-assisted design as one possible future direction without methodological detail [2]. Kamble (2026) reviewed multiscale modeling of effective permeability in polymer-based magnetic composites, covering classical effective medium theory, finite-element phase-field simulation, and the ML frontier, but the scope stayed limited to permeability as a single physical quantity [53]. Nguyen et al. (2025) gave a perspective on absorption-dominant EMI shielding from a design-strategy viewpoint, with limited AI coverage [54].

In these reviews, AI/ML is either briefly mentioned as an auxiliary tool or restricted to a specific physical quantity or material system. A review that starts from the AI/ML methodology itself and covers its application across the full technology stack of absorbing materials is still missing. In particular:

- ML models used in this field have not been classified and compared systematically, from traditional RF/XGBoost to recent Transformer and generative models;
- The application paradigms of AI in absorbing materials, from forward prediction to inverse design and from physics-informed modeling to explainability, have not been summarized in a panoramic way;
- Frontier paradigms such as large language models (LLM) and AI Agents have not been discussed for this field.

To fill these gaps, this review takes the AI methodology as the main viewpoint and surveys machine learning in electromagnetic wave absorbing materials. The rest of the paper is organized as follows. Section 2 introduces the core physical mechanisms of electromagnetic wave absorption and the mapping from physical equations to ML objective functions. Section 3 classifies the major AI/ML model paradigms used in this field, including classical machine learning, deep learning, generative models, large language models, AI Agents, physics-informed models, and optimization algorithms. Section 4 summarizes eight application paradigms, including forward property prediction, high-throughput screening, inverse generative design, electromagnetic-parameter decoupling, physics-informed modeling, explainability, multi-objective optimization, and data augmentation. Section 5 discusses future opportunities and challenges, including data standardization, foundation models, autonomous laboratories, LLM-assisted design, digital twins, multiscale modeling, and engineering-scale validation. Section 6 concludes the review.

### 1.5. Scope and Workflow Taxonomy

In this review, we use “absorber research” as an umbrella term covering both material-centric absorbing composites and structure/metasurface-based absorbers, while explicitly distinguishing their descriptors, datasets, and validation workflows. The workflow taxonomy for ML-assisted absorber design is summarized in Table 1.

## 2. Fundamentals of Electromagnetic Wave Absorption and the Data-Driven Paradigm

### 2.1. Core Physical Concepts

This section keeps the minimum set of concepts that link directly to the data-driven methods in later sections; more complete physical reviews can be found in [2,3,4,5,53].

#### 2.1.1. Reflection Loss: The Key Performance Metric

Absorber performance is evaluated through transmission line theory. When an electromagnetic wave travels from free space and impinges normally on an absorber backed by a perfect electric conductor (PEC), the reflection loss (*RL*, in dB) reads [55]:

$$RL=20\log_{10}\left|\frac{Z_{in}-Z_{0}}{Z_{in}+Z_{0}}\right|,\;{\;Z}_{in}=Z_{0}\sqrt{\frac{\mu_{r}}{\varepsilon_{r}}}\tanh\left[j\frac{2\pi fd}{c}\sqrt{\mu_{r}\varepsilon_{r}}\right]$$

Here, $Z_{0}=377\;\Omega$ is the wave impedance of free space, $\varepsilon_{r}=\varepsilon^{\prime }-j\varepsilon^{\prime \prime }$ is the complex permittivity, $\mu_{r}=\mu^{\prime }-j\mu^{\prime \prime }$ is the complex permeability, d is the layer thickness, and f is the frequency. *RL* < −10 dB (i.e., ≥90% energy absorbed) is the common threshold for effective absorption. In many data-driven studies, ML models predict the electromagnetic parameters ($\varepsilon_{r},\;\;\mu_{r}$) or directly predict absorption performance. When $\varepsilon_{r},\;\;\mu_{r}$ and the thickness d are known, the transmission-line equation is then used to calculate *RL* and evaluate the absorber design [7,41].

Although the minimum reflection loss is widely reported, it only describes the deepest absorption at a specific frequency and thickness. Therefore, this review does not use *RL_min_* as a standalone criterion. Instead, effective absorption bandwidth, matching thickness, and multi-objective performance are emphasized where available.

#### 2.1.2. Impedance Matching and Attenuation Constant

The design goal reduces to balancing two quantities that pull in opposite directions. Impedance matching decides whether the wave can enter the material: the closer the normalized impedance Z= |${\;Z}_{in}$/$Z_{0}$|is to 1, the smaller the reflection at the air–material interface. The attenuation constant α decides whether the wave, once inside, can be dissipated efficiently:*

$$\alpha =\frac{\sqrt{2}\pi f}{c}\sqrt{(\mu^{\prime \prime }\varepsilon^{\prime \prime }-\mu^{\prime }\varepsilon^{\prime })+\sqrt{(\mu^{\prime \prime }\varepsilon^{\prime \prime }-\mu^{\prime }\varepsilon^{\prime }{)}^{2}+(\mu^{\prime }\varepsilon^{\prime \prime }+\mu^{\prime \prime }\varepsilon^{\prime }{)}^{2}}}$$

Attenuation comes from two groups of mechanisms: dielectric loss (Debye relaxation, interfacial polarization, conduction loss), governed by the dielectric loss tangent $tan\delta_{\varepsilon }=\varepsilon^{\prime \prime }/\varepsilon^{\prime }$, and magnetic loss (eddy-current loss, natural and exchange resonance, domain-wall resonance), governed by the magnetic loss tangent $tan\delta_{\mu }=\mu^{\prime \prime }/\mu^{\prime }$. The usual moves to raise α (e.g., adding more conductive filler) push $\varepsilon^{\prime }$ up and move Z away from 1, and vice versa. AI-based optimization searches for a Pareto front between impedance matching (Z≈1) and high attenuation, a high-dimensional multi-objective problem that fits ML well [56].

### 2.2. Physical Limits and Why AI Is Needed

Absorber performance is bounded by hard physical laws. Two classical limits explain why traditional parameter tuning now gives diminishing returns.

The Rozanov thickness–bandwidth limit can be expressed as

$$\int_{0}^{\infty }ln|\frac{1}{\Gamma (\lambda )}|d\lambda \leq 2\pi^{2}\mu_{s}d$$

where Γ(λ) is the reflection coefficient, $\mu_{s}$ is the static permeability, and d is the absorber thickness. The physical meaning is that thin and broadband conflict by nature: strong low-frequency absorption needs either enough thickness or a large static permeability $\mu_{s}$.

The Snoek limit [9] for ferromagnetic materials is given by

$$(\mu_{s}-1)f_{r}=\frac{2}{3}\gamma M_{s}$$

where $f_{r}$ is the ferromagnetic resonance frequency, γ is the gyromagnetic ratio, and $M_{s}$ is the saturation magnetization. The product of the static permeability and the resonance frequency is a material constant. Traditional ferrites, therefore, struggle to keep both a large $\mu_{s}$ and a high working frequency in the GHz band.

Facing these limits, traditional designs stay within known material configurations and tune parameters such as filler ratio, layer thickness distribution, and impedance gradient, with shrinking marginal gain. Generative AI takes a different route: instead of sticking to known topologies, it creates new material configurations in a much larger structural space, such as free-form metasurface patterns [57] or asymmetric gradient composite foams [58]. These AI-assisted designs do not violate the Rozanov or Snoek limits. Rather, they help researchers search for structures closer to practical optima within physical constraints, or explore architectures whose assumptions differ from those of classical bounds.

### 2.3. From Physical Equations to ML Objective Functions

Data-driven research on absorbing materials groups into three basic problem types. Firstly, the forward problem aims to predict absorber performance from given material and structural parameters. In this task, the input usually includes the real and imaginary parts of permittivity and permeability, absorber thickness, and operating frequency, while the output is the reflection loss or effective absorption bandwidth. In practice, machine learning models may either directly predict the reflection loss curve or first predict the frequency-dependent electromagnetic parameters, which are then used in transmission-line theory to calculate the absorption response. The training data are typically obtained from vector network analyzer measurements or full-wave electromagnetic simulations such as CST and HFSS. Once trained, the machine-learning surrogate can evaluate new designs within milliseconds, greatly reducing the dependence on repeated experiments and time-consuming simulations [7,41].

The inverse problem aims to identify feasible material or structural designs that satisfy a desired absorption target. Unlike forward prediction, inverse design is inherently more challenging because the relationship between performance and structure is not one-to-one. The same reflection-loss curve may be achieved by multiple combinations of permittivity, permeability, thickness, composition, and microstructure [57]. Therefore, conventional single-output models often fail to capture the diversity of valid solutions. Generative models, such as variational autoencoders, generative adversarial networks, and mixture density networks, provide a more suitable solution by learning the distribution of feasible designs. They can generate multiple physically reasonable and structurally diverse candidates for the same target performance, enabling broader exploration of the absorber design space.

Embedding physical constraints handles the fact that experimental data on absorbers are usually scarce (a few hundred to a few thousand samples) and noisy [59]. Common moves include physics-informed loss (PINN) [60,61,62,63,64,65,66,67,68,69,70,71,72,73,74,75], which adds the transmission line equation, Maxwell’s equations, or the Kramers–Kronig causality relation as regularization terms; equivalent-circuit-model (ECM) guidance, where the ML model predicts RLC parameters and the ECM then computes the S-parameters analytically; and multi-fidelity modeling, which combines a small number of high-fidelity full-wave simulations with a large number of low-fidelity surrogate evaluations to improve sample efficiency.

## 3. AI/ML Model Paradigms and Tool Spectrum

The toolbox of machine learning and artificial intelligence used in electromagnetic wave absorbing materials keeps expanding. This section surveys the AI/ML models that have already entered this field, or that show clear potential value for it, and groups them into seven categories: classical machine learning, deep learning, generative models, large language models, AI Agents and self-driving labs, physics-informed and hybrid models, and optimization algorithms [76,77,78]. Table 2 at the end of the section summarizes the representative AI/ML works in the electromagnetic wave absorbing material field.

### 3.1. Classical Machine Learning

Classical ML models build fast surrogates and run feature-importance analysis on small-to-medium tabular datasets that link composition, processing, and properties. They do not need the input reshaped into images or sequences, train on a CPU within seconds to minutes, and offer good interpretability. For these reasons, they remain the most accessible and most frequently reported AI tools in this field.

**Figure 2:**
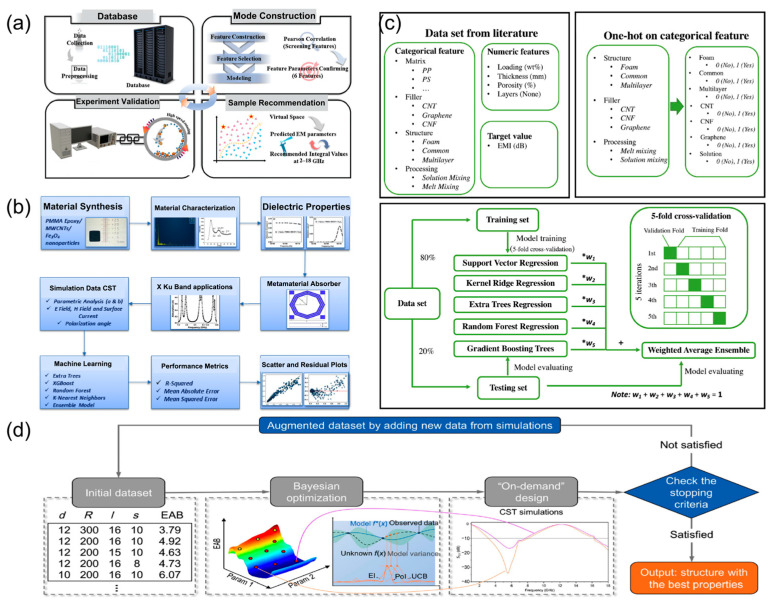
Representative machine-learning-assisted workflows for electromagnetic functional materials: (**a**) a closed-loop forecasting system for electromagnetic wave absorbent discovery, integrating database construction, feature screening, model training, sample recommendation, and experimental validation; (**b**) an ML-assisted metamaterial absorber design route combining nanocomposite synthesis, dielectric characterization, CST simulation, model prediction, and performance evaluation; (**c**) an ensemble-learning framework for EMI shielding prediction based on literature-derived datasets, feature encoding, model training, cross-validation, and weighted model fusion; and (**d**) a Bayesian active-learning strategy for broadband metasurface optimization, where simulations, candidate selection, dataset augmentation, and stopping-criterion checking are iteratively coupled to identify the optimal structure.

Random forest (RF) appears the most often and usually serves as a surrogate that predicts dielectric and absorbing properties from composition. Zhang et al. [41] used RF in their DCPRO system to predict the complex permittivity of flexible graphene-based composites, and used the expanded database to design a two-layer impedance-gradient absorber, reaching an effective absorption bandwidth (EAB) of 3.29–18 GHz and a minimum reflection loss of −56.08 dB. As shown in Figure 2a, Che et al. [7] built the MLFS framework around RF combined with pattern-recognition algorithms, ran high-throughput screening in a high-dimensional carbonyl iron/Fe_3_O_4_ design space, and raised the absorption performance by 207% and the bandwidth by 360%.

Gradient boosting trees (XGBoost/LightGBM/CatBoost) kick in when the feature dimension is high and the prediction accuracy needs to be pushed further. As presented in Figure 2b, Jain et al. [79] compared CatBoost, Extra Trees, XGBoost, random forest, and KNN in parallel and fused them by weighted ensembling, reaching R^2^ > 0.99 in absorbance prediction and applying the model to engineering-level inverse design. No single model wins across the board in the strongly nonlinear feature–property relations of absorbing materials, and ensembling stays robust. As illustrated in Figure 2c, Shi et al. [56] used a weighted ensemble of five heterogeneous base models (RF, XGBoost, SVR, etc.) for the EMI shielding effectiveness of carbon-based polymer nanocomposites, beating any single model and pairing the prediction with SHAP and PDP/ICE analyses to extract design rules.

Support vector regression (SVR) and Gaussian process regression (GPR) fit small-sample scenarios where experimental data are scarce and the model needs to provide an uncertainty estimate. The built-in uncertainty of GPR is its most useful feature in the absorbing-material context, since it directly drives Bayesian optimization with active sampling (see Section 3.7). Pure SVR/GPR as the final predictor is rarely reported here, but using them as the kernel model inside Bayesian active learning has started to appear: Figure 2d shows that Liu et al. [80] used GPR as the surrogate to drive active sampling in the design of an ITO-based broadband metasurface absorber, and matched the result of thousands of grid-sweep simulations with only about 200 simulation calls.

### 3.2. Deep Learning

Deep learning models handle inputs that carry spatial structure (metasurface patterns, SEM images), sequential structure (frequency-dependent $\varepsilon_{r}/\mu_{r}$ spectra), or that need a high-dimensional nonlinear mapping. Once the dataset reaches thousands to tens of thousands of samples, or once the input is a high-dimensional structured object, deep learning outperforms classical ML.

**Figure 3:**
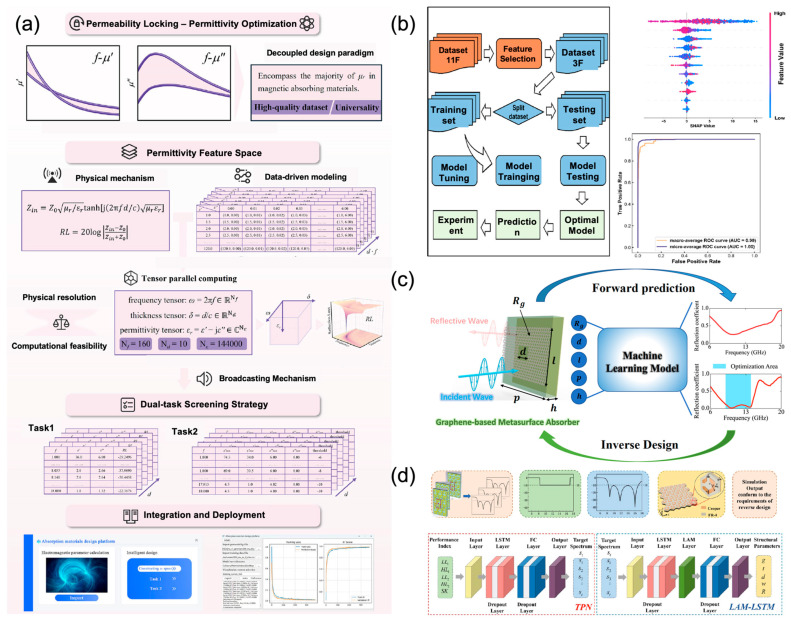
Representative machine-learning-enabled strategies for electromagnetic wave absorber and metasurface design: (**a**) neural-network-based permittivity engineering for magnetic absorbers through permeability locking, tensorized parameter-space screening, and dual-task optimization; (**b**) ML-guided interface engineering of MXene films, integrating feature selection, model training, SHAP-based interpretation, and experimental validation; (**c**) forward prediction and inverse design of graphene-based metasurface absorbers using a lightweight ML model linking structural/electrical parameters with reflection spectra; and (**d**) LAM-LSTM-assisted inverse design of hexagonal honeycomb metasurfaces, where target spectra are converted into optimized structural parameters for simulation validation.

Deep neural networks (DNN/MLP) form the most general deep architecture and often serve as high-capacity surrogates that predict frequency-dependent electromagnetic parameters from composition and structure. As shown in Figure 3a, Liu et al. [81] proposed a permeability-locking, permittivity-optimization two-task strategy based on DNN: the high-dimensional permittivity feature space is first built from tensor electromagnetic theory, and the DNN then predicts the matching performance and screens feasible solutions in one go, finally achieving an EAB of 5.1 GHz at 1.0 mm thickness and a reflection loss of −45.12 dB in a flake carbonyl iron/BaTiO_3_ system. It can be seen from Figure 3b that Zhou et al. [82] used DNN to guide the structural optimization of an MXene five-layer film with alternating sheets and hollow spheres, reaching a minimum reflection loss of −48.15 dB and an EAB of 5.84 GHz.

Convolutional neural networks (CNN) tackle spectrum prediction or inverse-design problems that take 2D topology patterns or microscopy images as input. One typical line of work feeds the metasurface pattern directly as an image and predicts the absorption spectrum: Figure 3c illustrates that Chen et al. [83] proposed a lightweight CNN that uses transposed convolution layers for both forward prediction and inverse design of graphene-based microwave metasurfaces, and treats the tunable conductivity of graphene as an extra design degree of freedom for multi-spectrum customizable absorption. Another line feeds SEM/TEM images directly into a CNN to learn the mapping from microscopic morphology to macroscopic absorbing performance, and pairs it with explainability methods such as Eigen-CAM to expose how pores and interfaces contribute to the loss.

Recurrent networks (RNN/LSTM/GRU) suit end-to-end modeling of one-dimensional sequence data such as spectra. As depicted in Figure 3d, Sun et al. [84] proposed LAM-LSTM (LSTM with a local attention mechanism) for the inverse design of a hexagonal close-packed metasurface absorber: a target prediction network first generates the desired S_11_ curve, and LAM-LSTM then recovers the geometry. The model reaches an MSE below 1.6 × 10^−4^ on the validation set, and the experiment confirms an absorption bandwidth of 8–17 GHz.

Transformers and attention mechanisms handle long-range dependency modeling and spectrum prediction or inverse design under multimodal conditions. The LEFormer proposed by Dong et al. [57] pairs convolutional layers with a Transformer encoder and serves as the forward predictor inside the Chimeric VAE framework. With only 2585 training samples, it predicts the absorption spectrum of free-form metasurface absorbers accurately, and filters physically invalid generations on the fly inside the generative loop.

### 3.3. Generative Models

Generative models address the one-to-many problem in inverse design. Unlike the models above, which learn a single-valued mapping, generative models learn the data distribution p(x) or the conditional distribution p(x|y), and sample multiple new feasible designs from the distribution.

**Figure 4:**
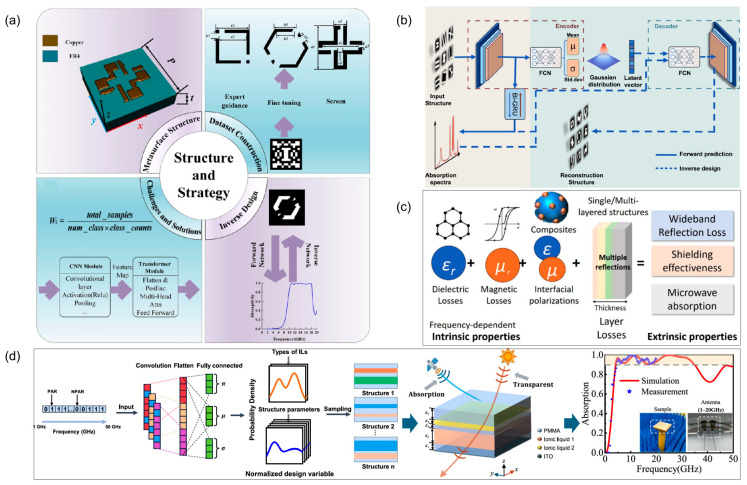
Representative deep-learning-based inverse design frameworks for electromagnetic absorbers and metasurfaces: (**a**) a Chimeric VAE–LEFormer strategy for free-form metasurface absorbers, integrating dataset construction, forward prediction, inverse generation, and small-sample learning solutions; (**b**) a CVAE-based multi-topology inverse design framework for terahertz metamaterial sensors, enabling probabilistic generation of diverse candidate structures from target spectra; (**c**) a physics-informed explainable learning framework linking intrinsic material parameters, loss mechanisms, and extrinsic absorption or shielding performance; and (**d**) an MDN-assisted inverse design route for transparent multilayer ionic-liquid absorbers, generating multiple feasible structures that satisfy broadband absorption targets.

Variational autoencoders (VAEs) are the most common generative model in the inverse design of absorbing materials. Figure 4a shows that Dong et al. [57] proposed the Chimeric VAE-LEFormer, which works as follows. A pretrained VAE and a secondary VAE share the decoder, and KL-divergence regularization gradually turns blurred initial patterns into clean metasurface structures, while the LEFormer forward predictor filters physically invalid generations online. With only 2585 training samples, the framework realizes inverse generation of free-form metasurfaces operating from 8 to 18 GHz.

Conditional VAE (CVAE) extends VAE by taking the target performance as an extra conditional input and generates the matching structure directly from a target absorption spectrum to enable controlled inverse design. As shown in Figure 4b, Sun et al. [87] proposed in 2026 a CVAE-based framework for multi-topology inverse design of THz metamaterial sensors. It builds an end-to-end bidirectional mapping between the response spectrum and the geometric topology, and generates several structurally diverse candidates for the same target spectrum. CVAE fits the one-to-many nature of electromagnetic inverse problems, and the same idea transfers directly to GHz absorbing metasurfaces.

Generative adversarial networks (GANs) work in scenarios where the generated structures need to be sharper than VAE outputs, while a less stable training process is acceptable. As depicted in Figure 4c, Narang et al. [85] proposed an explainable adversarial network that embeds explainability constraints into the generative decisions of a GAN for EMI shielding material design, combining GAN with XAI in this field at an early stage.

Diffusion models now lead image generation and, in principle, fit the one-to-many inverse problem, but in absorbing materials, they remain almost unexplored, leaving room for the next 2–3 years. Mixture density networks (MDNs) suit low-dimensional parametric inverse problems where the model needs to output multiple feasible solutions together with their probability weights. He and Zhao [43] used an MDN to design transparent broadband absorbers based on different ionic liquids: for a given target bandwidth, the model outputs several feasible combinations of ionic-liquid type and layer thickness, which are then verified by simulation, as illustrated in Figure 4d. The final transparent absorber reaches an absorption bandwidth of 4.18–34.9 GHz with an average visible-light transmittance of 76.5%.

### 3.4. Large Language Models (LLMs)

Large language models address scientific knowledge extraction, reasoning, and tool orchestration at the natural-language level [88,89,90,91,92,93,94,95,96,97,98,99,100,101,102]. Their direct engineering use in absorbing materials is still limited, but their potential value lies in three near-term tasks: extracting composition–processing–microstructure–property relations from the vast literature on absorbing materials, including filler type and content, processing history, morphology, $\varepsilon_{r}$(f), $\mu_{r}$(f), *RL*(f), EAB, and thickness; acting as the natural-language front end of an interactive material-design assistant that works together with inverse models such as VAE or MDN; and calling CST, HFSS, or COMSOL through APIs to enable natural-language-driven electromagnetic simulation workflows [103,104,105,106,107,108,109,110,111,112,113,114,115,116]. Three main bottlenecks remain: (i) the lack of a domain-specific LLM fine-tuned on electromagnetic-material data; (ii) the hallucination issue of general LLMs, which may produce material suggestions that look plausible but are physically wrong; and (iii) the fact that LLMs cannot perform numerical electromagnetic computation on their own, and need deep integration with professional simulators and physical models to become useful.

At the current stage, it is important to distinguish demonstrated LLM capabilities from future opportunities in absorber research. Demonstrated capabilities in the broader materials field mainly include scientific text mining, named-entity recognition, relation extraction, metadata cleaning, and construction of structured databases from the literature. For electromagnetic wave absorbing materials, the most realistic near-term use of LLMs is therefore not autonomous material invention, but automated extraction of absorber-specific variables from papers, including frequency-dependent $\varepsilon_{r}$(f), $\mu_{r}$(f), *RL*(f), EAB, thickness, composition, filler loading, morphology, processing conditions, and measurement metadata. These extracted records can then be normalized in units, checked for missing values, linked to raw figures or tables, and integrated into AI-ready databases. More ambitious tasks, such as LLM-driven inverse absorber design or autonomous decision-making, should be regarded as future opportunities and must be coupled with physical constraints, full-wave electromagnetic simulation, and experimental validation.

### 3.5. AI Agents and Self-Driving Labs

AI Agents automate the full research loop, which covers hypothesis generation, experiment or simulation, result analysis, and model update. Unlike traditional passive models, an Agent perceives, plans, acts, and reflects, and now sits at the frontier of AI research [17,62,70,71,72,76,77,78,117,118,119,120].

**Figure 5:**
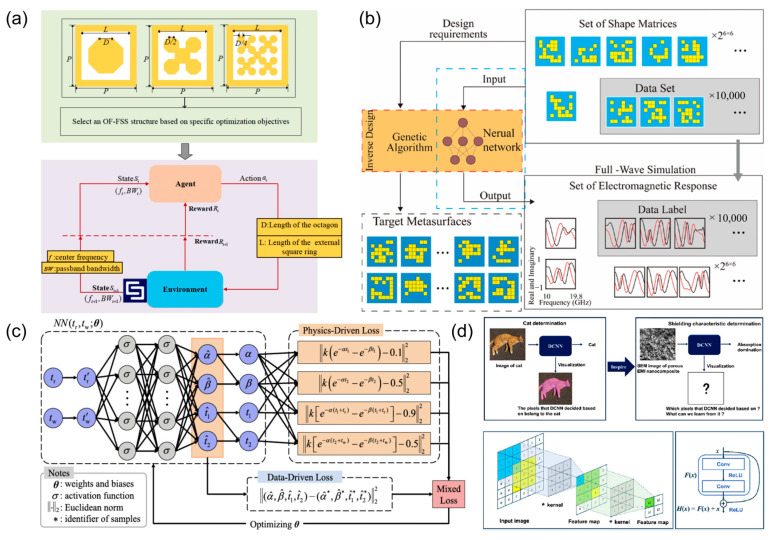
Representative AI-assisted design and interpretation methods for electromagnetic structures and functional materials: (**a**) physics-informed reinforcement learning for octagonal fractal FSS optimization, where an Agent adjusts structural parameters to meet target center-frequency and bandwidth requirements; (**b**) neural-network- and genetic-algorithm-driven inverse design of binary-coded metasurfaces for target electromagnetic responses; (**c**) physics- and data-co-supervised neural network for estimating electromagnetic pulse parameters through mixed data-driven and physics-driven losses; and (**d**) DCNN visualization for interpreting porous EMI nanocomposites, revealing the microstructural regions that dominate shielding behavior.

In the broader materials field, prototypes of self-driving labs have already appeared. The A-Lab at Berkeley (2023) [5] closed the full loop in which AI generates the hypothesis, robots carry out powder mixing and sintering, XRD performs the characterization, and AI then analyzes the results and designs the next round of experiments. Without human intervention, the system synthesized several novel inorganic solid-state materials. The ARES platform shows similar potential in accelerated material discovery. In the absorbing-material field, no full Agent-based autonomous experimental system has been reported, but key components are taking shape. As shown in Figure 5a, Dong et al. [86] combined reinforcement learning with physics-informed constraints for metasurface design decisions, as an early prototype of the decision engine of a future Agent system: the *RL* Agent picks actions (i.e., parameter updates) in the electromagnetic design space and learns the optimal policy from simulation-based reward signals. A natural multi-Agent architecture would split the work across four roles: a design Agent generates candidate structures, a simulation Agent calls tools such as CST or HFSS for electromagnetic verification, an experiment Agent controls robots to fabricate and test the key samples, and an analysis Agent interprets the results and updates the models, all working around a shared knowledge base to close the material-discovery loop.

A similar distinction is needed for AI Agents. Autonomous laboratories have been demonstrated in the broader materials and chemical sciences, but a full closed-loop autonomous laboratory for electromagnetic wave absorbing materials has not yet been reported. In the absorber field, the demonstrated components are still partial, such as ML surrogate models, Bayesian or evolutionary optimization, physics-informed reinforcement learning for structural search, and automated simulation workflows. Therefore, near-term Agent applications should focus on human-in-the-loop tasks, including literature retrieval, database construction, simulation-job preparation, parameter sweeping, candidate ranking, uncertainty checking, and report generation. Fully autonomous absorber-design Agents that can close the loop from hypothesis generation to fabrication, VNA testing, model updating, and mechanism interpretation should be framed as a long-term prospect rather than an established capability.

### 3.6. Physics-Informed and Hybrid Models

Physics-informed methods address the case where experimental data are scarce and a pure black-box model produces predictions that are not physically self-consistent. The core idea embeds known physical laws—Maxwell’s equations, the transmission line equation, and the Kramers–Kronig causality relation—as constraints in the training, so that predictions stay bounded by physics even in data-sparse regions [121].

In practical PINN implementation, these physical laws are usually introduced as additional terms in the loss function rather than only as post-processing criteria. For example, a neural network can be trained to predict frequency-dependent electromagnetic parameters or absorption responses, while the training loss is constructed as a combination of data-fitting error and physics-residual error. In absorber design, the predicted ($\varepsilon_{r}$), ($\mu_{r}$), thickness (d), and frequency (f) can be substituted into the transmission-line equation to calculate ($Z_{in}$) and (*RL*). The deviation from measured or simulated data forms the data loss, whereas violation of the transmission-line relation, Maxwell-equation residuals, Kramers–Kronig causality, passivity, or boundary conditions can be used as physics-informed penalties. In this way, PINNs constrain the model to learn not only statistical correlations from limited data but also physically consistent relationships among electromagnetic parameters, absorber thickness, and absorption performance.

Physics-informed neural networks (PINNs) provide a representative framework for accelerating electromagnetic-field simulations by embedding governing physical equations into neural-network training. In the referenced work, a current-density-based PINN was developed to solve Poisson’s equation for electromagnetic-field prediction. Unlike conventional numerical solvers such as the finite difference method (FDM), finite element method (FEM), and discontinuous Galerkin method, which often require repeated computation when the excitation source or boundary condition changes, the proposed PINN learns the mapping from current-density-related inputs to the electric-potential or electric-field distribution. The network architecture consists of a deep neural network, automatic differentiation for evaluating PDE residuals, and a hybrid loss function that combines data loss, Poisson-equation residual loss, and electric-field constraint loss.

This strategy allows the model to preserve physical consistency while improving computational efficiency and adaptability. Gao et al. [122] validated the method in two representative electromagnetic scenarios: electromagnetic pulses generated by laser–target interaction and electric-field calculation in field–circuit coupling problems. The results showed that the PINN model can achieve prediction accuracy comparable to traditional FDM solutions while greatly reducing the computational cost. For example, in two-dimensional simulations, the FDM required 102.21 s for 10,000 models, whereas the PINN model required only 0.18 s, with average relative errors remaining below 0.16% across different scenarios and random seeds. Therefore, this work demonstrates that physics-informed learning can serve as a fast, physically constrained surrogate for electromagnetic-field simulation, providing a promising tool for absorber and electromagnetic-material design where repeated field calculations are computationally expensive. However, the effectiveness of such physics-informed models depends on whether the embedded physical assumptions, such as homogeneity, normal incidence, effective electromagnetic parameters, and boundary conditions, are consistent with the real absorber system. Therefore, PINN-based predictions should still be validated by external test data, full-wave simulations, or experimental measurements.

### 3.7. Optimization Algorithms (Usually Coupled with ML Models)

Optimization algorithms do not belong to machine learning, but in data-driven research on absorbing materials, they almost always pair with ML surrogate models, so we cover them here. The ML surrogate provides millisecond-level performance evaluation, while the optimization algorithm searches for the Pareto optimum in a wide design space.

Genetic algorithms (GA) handle global combinatorial optimization in discrete or mixed parameter spaces. They mimic the selection–crossover–mutation process of natural evolution, and once coupled with a surrogate, the population size can be enlarged considerably. It can be seen from Figure 5b that Tao et al. [123] coupled a neural-network surrogate with GA for the inverse design of multilayer absorbing sandwich structures. The NN evaluates the absorbing performance of every individual at the millisecond level, while GA jointly optimizes the number of layers, the layer thicknesses, and the material choice. The final non-uniform gradient design delivers clearly broader bandwidth across the full band than a conventional single-layer uniform reference [124].

Particle swarm optimization (PSO) suits fast global optimization in continuous parameter spaces. Each particle represents a candidate parameter set and updates its position based on its own historical best and the global best. As depicted in Figure 5c,d, Zhou and Li [42] coupled an RF surrogate with PSO, took the volume fraction of each layer as the design variable and the total absorption bandwidth as the objective, and optimized the nonlinear gradient distribution of a multilayer Octet lattice. The optimized design clearly beats the conventional uniform and linear-gradient designs.

Bayesian optimization (BO) addresses optimization problems where each simulation or experiment costs a lot and the available sample budget is limited. BO uses GPR as the surrogate and balances exploitation and exploration through an acquisition function, picking the most informative next evaluation point at each step. Liu et al. [80] applied Bayesian active learning to broadband metasurface absorber design and obtained a lightweight, polarization-insensitive ITO-based broadband absorber with only a few hundred electromagnetic simulations, closing the BO loop in this field. Topology optimization and the adjoint method rely on gradient information for continuous-parameter optimization. When combined with differentiable FDTD/FDFD frameworks, they integrate with neural networks for end-to-end gradient back-propagation and form an important direction for future coupling with generative models.

Because different absorber-design tasks involve different data structures, no single ML model is universally optimal. Model selection should depend on the data type, sample size, descriptor form, target property, and validation requirement. Table 3 summarizes practical guidance for selecting suitable ML models and validation protocols for common tasks in electromagnetic wave absorbing materials.

## 4. AI/ML Application Paradigms in Electromagnetic Wave Absorbing Materials

Section 3 organized AI/ML by model type. This section reorganizes the same field by application paradigm, and groups the AI work in electromagnetic wave absorbing materials into eight paradigms—forward property prediction, high-throughput screening and combinatorial optimization, inverse structure and composition generation, intelligent retrieval and decoupling of electromagnetic parameters, physics-informed and hybrid modeling, explainability and mechanism discovery, multi-objective and multifunctional co-optimization, and data augmentation and few-shot learning. Table 4 at the end of the section summarizes the eight paradigms together with representative works.

To avoid a purely descriptive listing of models and case studies, this section further provides a critical synthesis for each application paradigm. For each paradigm, we discuss not only representative works but also its suitable data types, descriptor requirements, model-selection logic, typical failure modes, uncertainty sources, and recommended validation strategies. This perspective is important because different AI methods are not interchangeable in absorber research. A model that works well for tabular composition–property prediction may fail for spectral sequence learning or metasurface topology generation, while a generative inverse-design model may produce visually reasonable structures that are physically invalid without forward electromagnetic verification. Therefore, the following subsections emphasize both what each paradigm can achieve and under what conditions its conclusions should be trusted.

### 4.1. Forward Property Prediction

**Figure 6:**
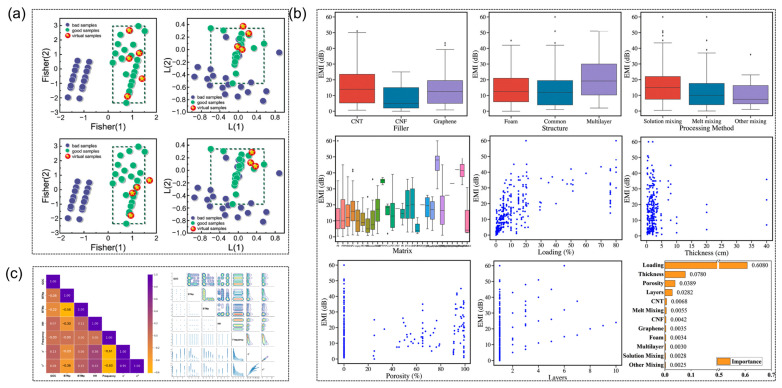
Representative data-analysis and feature-mining results in machine-learning-assisted electromagnetic material design: (**a**) high-throughput screening and inverse projection for distinguishing good, bad, and virtual microwave-absorbent candidates; (**b**) statistical distributions and feature-importance analysis of EMI shielding datasets, revealing the influence of filler type, structure, processing method, loading, thickness, porosity, and layers; and (**c**) correlation and pairwise feature analysis for flexible graphene-based absorptive composites, clarifying relationships among processing parameters, frequency, and electromagnetic responses.

Forward property prediction replaces expensive experiments or full-wave simulations with a trained surrogate model and finishes a single performance evaluation in milliseconds. As shown in Figure 6a, the inputs cover composition (filler type and content, element ratio), processing parameters (temperature, time, pressure) and structural parameters (thickness, layer number, porosity); the outputs cover the complex permittivity $\varepsilon_{r}$, the complex permeability $\mu_{r}$, the reflection loss (*RL*), the effective absorption bandwidth (EAB) or the EMI shielding effectiveness (SE) [7].

Che et al. [7] built MLFS as an early example of this paradigm. It uses random forest as the core predictor, with the composition and processing parameters of carbonyl iron/Fe_3_O_4_ composites as input and *RL* and EAB as output, and adds a pattern-recognition step to project high-performing candidates back into the design space. The final selected material raises the maximum absorption efficiency by 207% and the effective bandwidth by 360%, with a prediction correlation coefficient of 0.9844. It can be seen from Figure 6b that Zhang et al. [41] extended this idea in their DCPRO system in Chemical Engineering Journal by chaining forward prediction with impedance-gradient design: RF first predicts the complex permittivity from the processing parameters, and a two-layer structure with a low-impedance matching layer and a high-loss layer is then built on top, keeping *RL* < −10 dB across a 3.29–18 GHz band and reaching a minimum reflection loss of −56.08 dB. As illustrated in Figure 6a, Shi et al. [56] reported a weighted-average ensemble that fuses five heterogeneous base models (RF, XGBoost, SVR, KNN, GBDT) for the EMI SE of carbon-based polymer nanocomposites. The ensemble outperforms any single model, and the added use of SHAP, PDP and ICE turns the surrogate from a pure performance predictor into a tool that helps researchers extract design rules.

Forward prediction is the most mature and lowest-barrier paradigm in this field. Its accuracy depends on the coverage of the training data. It can only evaluate given candidates and cannot actively propose new ones; the screening of Section 4.2 closes the loop.

A critical point for forward prediction is that model performance is often dominated by the quality and physical relevance of input descriptors rather than by the algorithm itself. For tabular datasets, descriptors such as filler content, thickness, density, processing temperature, frequency, real and imaginary permittivity, permeability, porosity, and layer number should be reported consistently. Classical models such as RF, XGBoost, and SVR are usually reliable for small-to-medium datasets, but they can fail when the training and test sets share highly similar samples, when frequency points from the same sample are randomly split into both training and test sets, or when the model is asked to extrapolate to a new material family. Therefore, sample-level splitting, external test-set validation, and comparison with transmission-line calculations are more meaningful than random point-wise splitting alone. Uncertainty estimation should also be reported when the model is used for design recommendation rather than simple interpolation.

### 4.2. High-Throughput Screening and Combinatorial Optimization

High-throughput screening and optimization locates Pareto-optimal solutions in a candidate space of millions, by using a trained ML forward model as a fast fitness evaluator and coupling it with global search strategies such as genetic algorithms, particle swarm optimization, or Bayesian optimization [7,42].

**Figure 7:**
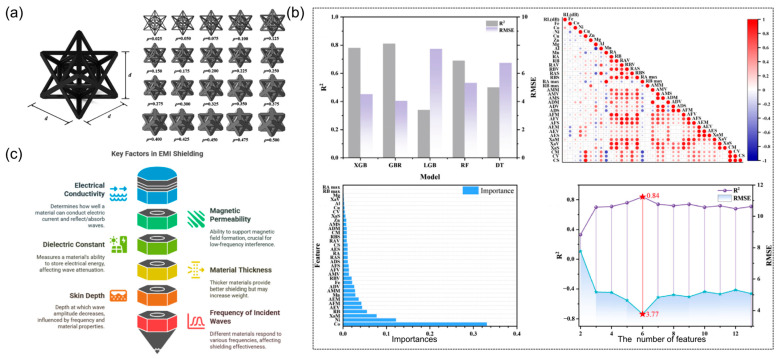
Representative machine-learning and mechanism-guided analyses for electromagnetic wave absorption and EMI shielding materials: (**a**) octet-lattice unit cells with tunable volume fractions for lightweight microwave-absorbing structure optimization; (**b**) model comparison, correlation analysis, feature-importance ranking, and descriptor selection for high-entropy spinel microwave absorbers; and (**c**) key physical factors governing EMI shielding, including conductivity, permittivity, permeability, thickness, skin depth, and operating frequency.

On top of forward prediction, the MLFS system of Che et al. [7] also realizes a complete virtual screening loop: the RF surrogate evaluates far more composition-and-processing combinations of carbonyl iron/Fe_3_O_4_ than would ever be feasible experimentally, and pattern recognition then extracts composition–performance patterns from the high-performing subset for human-guided experimental verification. As illustrated in Figure 7a, the RF + PSO joint optimization framework proposed by Zhou L. [42] fits continuous parameter spaces. They first run full-wave simulations on Octet-lattice unit cells with different volume fractions and retrieve effective electromagnetic parameters from the S-parameters; an RF surrogate then maps volume fraction to the effective parameters; and finally, PSO searches globally with the volume fractions of all layers as variables, the total absorption bandwidth as the objective, and the total volume fraction as a constraint. The optimum shows a non-linear volume-fraction gradient and clearly outperforms the uniform or linear gradients that human intuition tends to pick. As shown in Figure 7b, Liu et al. [125] and Singh et al. [126] further pushed ML surrogates to high-entropy spinel ferrites and high-entropy multi-element systems, where the combinatorial space is so vast that traditional trial-and-error is infeasible and ML surrogates remain the only practical route for fast screening.

The main bottleneck of this paradigm is that the convergence of evolutionary algorithms is bounded by the surrogate’s accuracy near the boundary of the parameter space. It can be seen from Figure 7c that replacing pure evolutionary strategies by Bayesian optimization, which uses GPR uncertainty for active exploration, is the main improvement direction [127].

For high-throughput screening, the main risk is that the optimization algorithm may overexploit errors of the surrogate model. Evolutionary algorithms or PSO can easily push the search toward the boundary of the descriptor space, where the surrogate has low training-data support and high extrapolation uncertainty. As a result, the predicted optimum may be a numerical artifact rather than a physically meaningful absorber. To reduce this risk, the candidate pool should be constrained by physically meaningful ranges of composition, thickness, density, processing conditions, and fabrication tolerance. Bayesian optimization or active learning is preferable when each simulation or experiment is expensive, because uncertainty can guide the next sampling point. The best practice is to verify the top-ranked candidates by full-wave simulation or experiment, and to update the model iteratively with these newly validated samples.

### 4.3. Inverse Structure and Composition Generation

Inverse generation recovers a material structure or composition from a desired absorption performance. The core difficulty is the one-to-many mapping of electromagnetic inverse problems: the same target absorption spectrum can be realized by many different structure or composition combinations. A directly trained single-valued network averages over equivalent solutions and outputs physically invalid designs, so the methodological key uses generative models that learn the distribution of feasible solutions rather than a single mapping.

**Figure 8:**
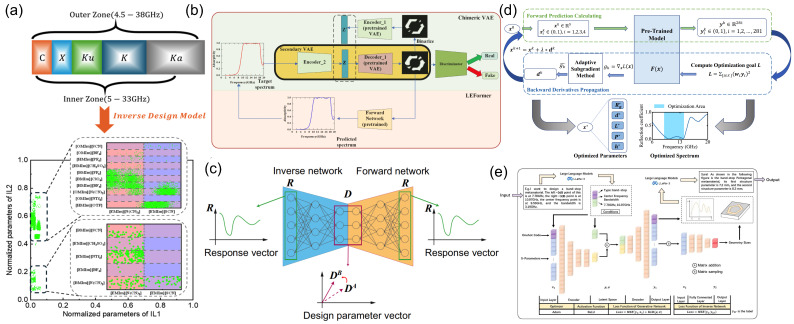
Representative inverse-design frameworks for electromagnetic absorbers and frequency-selective surfaces: (**a**) MDN-based inverse design of transparent ionic-liquid absorbers, identifying feasible material and thickness combinations for broadband absorption; (**b**) Chimeric VAE–LEFormer architecture for generating clear free-form metasurface patterns from target spectra with forward-network verification; (**c**) constrained tandem neural network for mapping desired microwave absorption responses to optimized multilayer metasurface parameters; (**d**) gradient-based inverse optimization of graphene metasurface absorbers using a pretrained forward model; and (**e**) LLM-assisted automatic FSS design, translating natural-language requirements into S-parameter targets and geometric parameters.

As shown in Figure 8b, Dong et al. [57] developed Chimeric VAE-LEFormer. It first pretrains a standard VAE on a large set of unlabeled metasurface patterns to learn a generic pattern prior, and then introduces a secondary VAE that shares the decoder. The encoder of the secondary VAE takes the target absorption spectrum as a conditional input, and KL-divergence regularization pulls its latent distribution toward that of the pretrained VAE, so that blurry initial patterns turn into clean structures step by step. The LEFormer forward predictor then performs fast electromagnetic verification on each generated structure and filters out physically invalid ones. With only 2585 training samples, the framework yields high-quality inverse generation of free-form metasurface absorbers operating from 8 to 18 GHz, with 99% pattern fidelity. The pretraining-and-finetuning strategy cuts the data needed for conditional generation, and pairing a forward predictor with the inverse generator builds a reliable physical-filter architecture.

For parametric structures, Sun et al. [84] proposed LAM-LSTM, in which a target prediction network first generates the desired S_11_ curve and an LSTM with a local attention mechanism then recovers the geometry; the validation MSE stays below 1.6 × 10^−4^ and the experiment confirms broadband absorption across 8–17 GHz. As shown in Figure 8a, He and Zhao [43] used a mixture density network (MDN) to design transparent broadband absorbers based on different ionic liquids: the MDN outputs several Gaussian components, each tied to a feasible combination of ionic-liquid type and layer thickness, and the final device reaches an absorption bandwidth of 4.18–34.9 GHz with a 76.5% visible-light transmittance. In Figure 8c, He et al. [128] proposed a tandem network, in which an inverse network generates structures and a pretrained forward network checks them; the loss is computed at the output of the forward network, which acts as a physical filter and bypasses the ill-posedness of direct inverse mapping. It can be seen from Figure 8d that other works include lightweight CNN by Chen et al. [83] that treats the tunable conductivity of graphene as an extra design degree of freedom, and the automated inverse design of frequency-selective surfaces by Shen et al. [129], as shown in Figure 8e.

The key advantage of inverse generation is its ability to address the one-to-many nature of absorber design, but this advantage also creates a validation challenge. A generated structure may match the target spectrum in the latent space or according to a learned surrogate, but it may still violate physical constraints, fabrication rules, or full-wave electromagnetic behavior. Typical failure modes include disconnected patterns, unrealistic minimum feature sizes, non-causal electromagnetic parameters, and solutions that perform well only under idealized boundary conditions. Therefore, generative inverse design should not be treated as a direct replacement for electromagnetic simulation or experiment. A reliable workflow should combine conditional generation, physical filtering, forward-network screening, full-wave simulation, and final experimental confirmation. For composition-based inverse design, additional constraints such as phase stability, processability, density, and environmental durability should be considered together with *RL* and EAB.

### 4.4. Intelligent Retrieval and Decoupling of Electromagnetic Parameters

**Figure 9:**
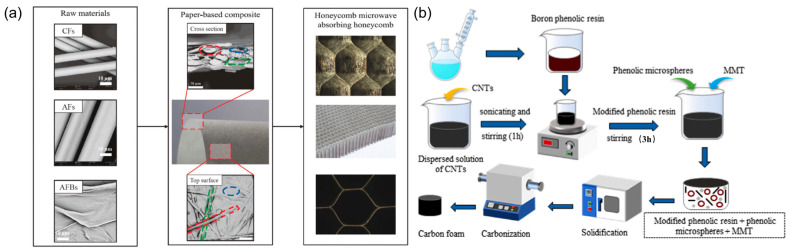
Representative structural design and fabrication routes for lightweight electromagnetic absorbing/shielding composites: (**a**) paper-based composite honeycomb absorbers fabricated from carbon fibers, aramid fibers, and aramid fibrids, showing the transition from raw fiber materials to composite paper and honeycomb microwave-absorbing structures; (**b**) CNT/MMT-reinforced carbon foam composites prepared through CNT dispersion, boron phenolic resin modification, microsphere/MMT addition, solidification, and carbonization for enhanced structural and EMI shielding performance.

This paradigm manipulates the intrinsic physical quantities $\varepsilon_{r}$ and $\mu_{r}$ that govern absorption, rather than the resulting *RL*. Liu et al. [81] proposed the Permeability-Locking and Permittivity-Optimization strategy. Magnetic absorbers have long been hindered by the strong coupling between $\varepsilon_{r}$ and $\mu_{r}$—changing the dielectric filler usually changes the magnetic response as well. Liu et al. first chose flake carbonyl iron with good magnetic loss as the magnetic matrix and locked $\mu_{r}$ as a constant; tensor electromagnetic theory was then used to compute *RL* and EAB in batch over a wide range of $\varepsilon^{\prime }$–$\varepsilon^{\prime \prime }$ combinations and built a high-dimensional permittivity feature space; a DNN was trained on this space to run a dual-task screening that locates both the optimum-absorption window and the effective-absorption window; and finally, the corresponding dielectric filler and ratio were chosen to synthesize a flake carbonyl iron/BaTiO_3_ composite, which reached an EAB of 5.1 GHz at 1.0 mm thickness and a reflection loss of −45.12 dB at 1.9 mm. The strategy provides a transferable route—parameter decoupling, intrinsic-space screening, and targeted experimental synthesis—that applies to any magnetic–dielectric composite system. As shown in Figure 9a, Xia et al. [130] proposed a method that splits the geometry-to-effective-parameter step from the effective-parameter-to-macro-property step and models them separately. In Figure 9b, Cao et al. [59] further applied a physics-enhanced approach to electromagnetic parameter extraction in absorbing-material structural analysis, confirming the accuracy advantage of the data-and-physics dual-driven strategy under limited samples.

This paradigm sits closer to physical essentials, but the inversion accuracy depends on the forward physical model: when assumptions of the transmission-line model (isotropy, uniform medium, etc.) do not match the real material, the inversion may deviate from the truth.

The decoupling and inversion of electromagnetic parameters provide more physical insight than directly predicting *RL*, but the reliability of this paradigm strongly depends on the quality of measured or retrieved parameters. Errors in VNA calibration, sample thickness, air gaps, NRW extraction, density normalization, and boundary-condition assumptions can be amplified during parameter inversion. In addition, effective permittivity and permeability are meaningful only under assumptions such as homogeneity, isotropy, and weak spatial dispersion, which may not hold for porous composites, anisotropic films, or patterned metasurfaces. Therefore, parameter-decoupling models should report the measurement protocol, frequency range, sample geometry, thickness, density, and extraction method. Cross-checking the retrieved parameters by recalculating *RL* through transmission-line theory is a useful validation step.

### 4.5. Physics-Informed and Hybrid Modeling

Physics-informed modeling prevents purely data-driven models from producing physically absurd predictions in data-sparse regions. The core idea embeds known physical laws—Maxwell’s equations, the transmission-line equation, the Kramers–Kronig causality relation, and equivalent-circuit models—as constraints in the ML training, so that the model stays physically reasonable and extrapolates well even with scarce, noisy or out-of-distribution data [59,86].

**Figure 10:**
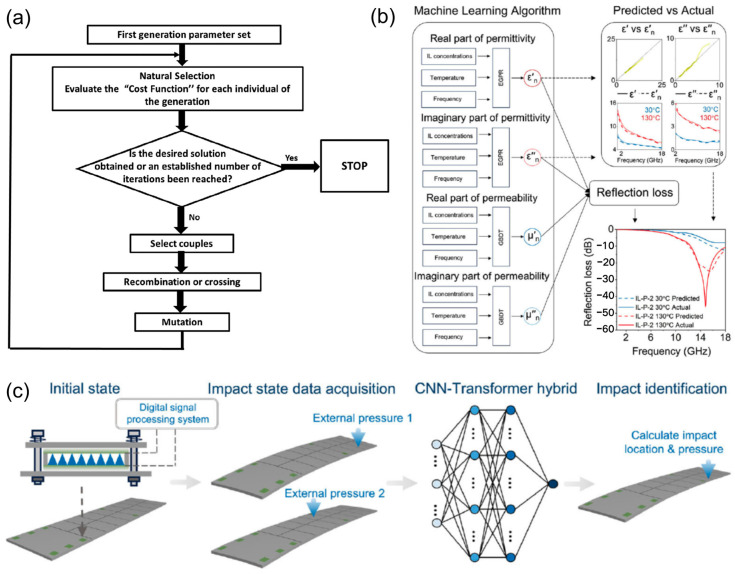
Representative optimization, prediction, and sensing workflows for electromagnetic materials and adaptive microwave surfaces: (**a**) genetic-algorithm optimization of magnetic permeability parameters through selection, crossover, mutation, and iterative cost-function minimization; (**b**) machine-learning prediction of temperature- and ionic-liquid-concentration-dependent electromagnetic parameters, followed by reflection-loss calculation and validation against measured data; and (**c**) CNN–Transformer-based structural health monitoring of adaptive microwave surfaces, where impact signals are collected and processed to identify the impact location and pressure.

Cao et al. [59] applied a physics-enhanced approach to absorber structural analysis and parameter extraction, with a loss function that pairs a data-fitting term with a physics-residual term that measures how much the prediction violates the transmission-line or Maxwell equations; even when the sample size is halved, the data-and-physics dual-driven model still beats the pure data-driven baseline. Dong et al. [86] embedded physics constraints into a reinforcement-learning framework for metasurface design decisions: at each step, the *RL* Agent picks an action that adjusts a geometric parameter, and the reward signal carries both the absorption-performance gain and a penalty for violating physical constraints (such as whether the geometry stays in the manufacturable range or whether the inter-cell coupling stays in the linear regime), which keeps the search inside the physically realizable subspace. As shown in Figure 10a, Boggi et al. [131] systematically studied causality constraints in the modeling of electromagnetic responses: the Kramers–Kronig relation requires a causality-determined integral relation between the real and imaginary parts of the permittivity, and any function violating it corresponds to a physically unrealizable material. Embedding such conditions as hard constraints or soft regularization in ML training prevents physically infeasible designs at the source.

This paradigm gains on data efficiency and prediction trustworthiness, at the price of higher implementation complexity, system-dependent forms of physical constraints (which limit transferability), and the risk that overly strict constraints suppress counter-intuitive but physically valid novel designs, like in Figure 10b,c [132].

Physics-informed models are most useful when data are scarce but reliable physical equations are available. However, physical constraints should be selected carefully. If the embedded equation does not match the real system, the model may become physically biased rather than physically reliable. For example, a transmission-line constraint is appropriate for a homogeneous metal-backed absorber under normal incidence, but it may be insufficient for anisotropic, multilayer, oblique-incidence, or strongly scattering structures. Similarly, Kramers–Kronig constraints improve causality consistency but require high-quality frequency-dependent electromagnetic parameters. The best practice is to treat physics-informed learning as a soft coupling between data and physics, compare it with a purely data-driven baseline, and analyze whether the physics term improves out-of-distribution prediction rather than only reducing training error.

### 4.6. Explainability and Mechanism Discovery

Explainability analysis extracts physical mechanisms from black-box models—it lets the model tell researchers which microscopic features (filler content, pore structure, interface morphology, element ratio, etc.) decide the absorbing performance, and pushes forward the basic understanding of the underlying physics rather than just the performance numbers [44,56].

**Figure 11:**
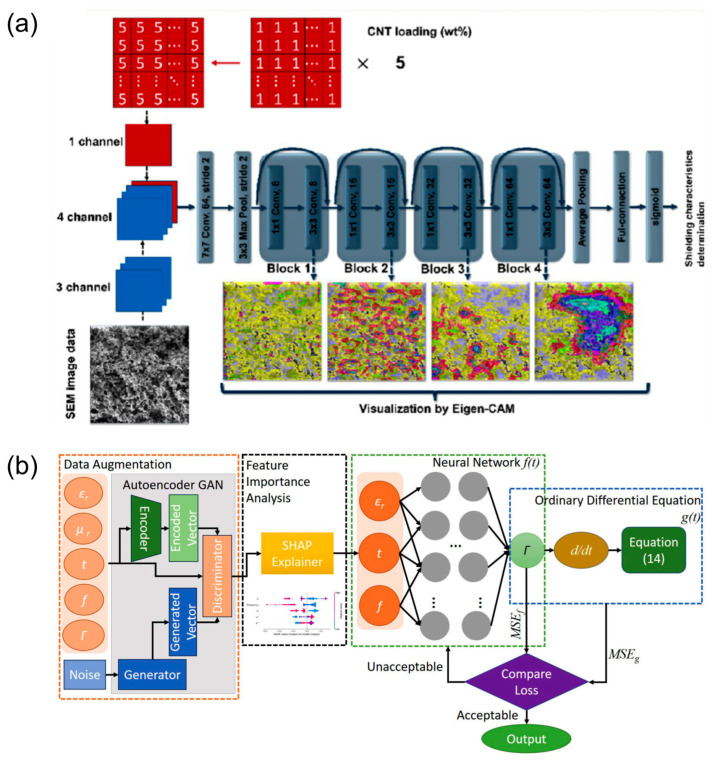
Representative explainable and physics-informed learning frameworks for electromagnetic functional materials: (**a**) ResNet-based DCNN visualization for porous CNT/PVDF EMI shielding composites, combining SEM images, CNT loading, shielding-characteristic classification, and Eigen-CAM heatmaps to reveal pore-related shielding mechanisms; and (**b**) XA-PINN framework integrating AGAN-based data augmentation, SHAP feature-importance analysis, and Riccati-equation-constrained neural networks to predict reflection behavior and interpret key electromagnetic parameters.

After building an EMI SE prediction model, Shi et al. [56] applied model-agnostic interpretability methods in series: SHAP quantifies the marginal contribution of each feature to SE, partial dependence plots (PDPs) shows the average effect of a single feature, and individual conditional expectation (ICE) further exposes individual differences hidden inside the PDP mean; together, they distill a set of design rules that researchers can use directly. As shown in Figure 11a, the follow-up DCNN + Eigen-CAM work by Shi et al. [44] pushed interpretability to the microscopic-image level: a modified deep residual network was trained on a large set of CNTs/PVDF porous-composite SEM images to map morphology directly to EMI SE, and Eigen-CAM was then used to overlay the CNN decision regions on the original SEM images as a heat map. The visualization quantifies an effect that traditional macroscopic porosity statistics had averaged away—shallow pores contribute much less to absorption than deep pores; the 30 wt% CNTs sample reported in the same work reached an EMI SE of 105 dB. As shown in Figure 11b, Narang et al. [85] embedded explainability analysis into the generative loop of a GAN so that the network explains its own design decisions while producing EMI shielding structures, while Arya et al. [133] developed a SHAP-based interactive GUI that wraps the analysis behind a graphical interface and lowers the entry barrier for materials researchers.

A key limitation of explainability methods is that they reveal statistical associations rather than direct causality. SHAP, PDP, ICE, and CAM-based visualization can identify important descriptors or image regions, but the highlighted features may be correlated with hidden variables such as processing route, density, sample thickness, or measurement frequency. Therefore, explainability results should be interpreted together with physical knowledge and controlled experiments. For tabular datasets, correlated descriptors should be checked before drawing conclusions from feature importance. For image-based models, CAM heatmaps should be compared with microstructural statistics such as pore size, connectivity, interfacial area, and filler distribution. In absorber research, explainability is most valuable when it generates testable physical hypotheses rather than when it is used only to decorate a black-box prediction.

### 4.7. Multi-Objective and Multifunctional Co-Optimization

**Figure 12:**
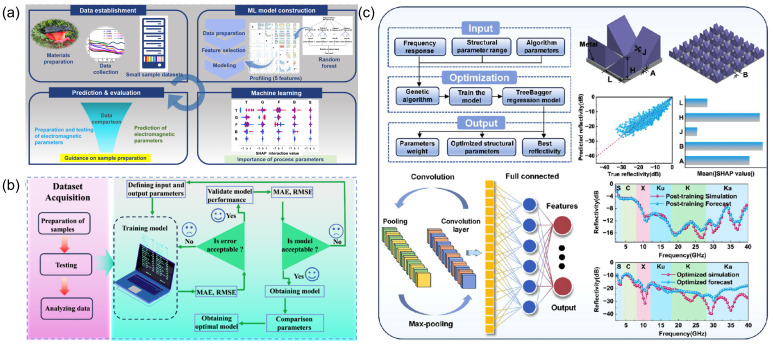
Representative machine-learning-assisted design workflows for multifunctional electromagnetic wave absorbing and EMI shielding materials: (**a**) ML-guided design of graphene-based aerogels, integrating data establishment, feature selection, random-forest modeling, SHAP interpretation, and electromagnetic-parameter prediction; (**b**) dataset-driven model training and validation workflow for absorption-dominant asymmetric gradient composite films, using error metrics to identify an optimal predictive model; and (**c**) optimization of bionic wedge-structured absorbers through genetic algorithms and regression models, linking structural parameters with broadband reflectivity prediction and performance enhancement.

Multi-objective and multifunctional co-optimization handles absorbing materials that must satisfy several conflicting goals at once—absorption bandwidth versus thickness, absorption strength versus structural weight, EMI shielding versus mechanical load and thermal conductivity, and so on. The task uses ML/AI to find the Pareto front in the multi-dimensional objective space, like in Figure 12b [134,135].

As shown in Figure 12a, Wang et al. [135] worked on multifunctional composites and put absorbing performance, mechanical strength and thermal conductivity into a single optimization framework; an ML surrogate then searches the multi-objective space and outputs a Pareto front for designers to pick from according to the application scenario. The RF + PSO framework of Zhou and Li [42] takes the layer-wise volume fractions as variables, the maximum absorption bandwidth as the objective, and the total mass as a constraint, and the resulting non-linear gradient structure clearly outperforms uniform or linear gradients under the same weight budget. The DCPRO system of Zhang et al. [41] uses a two-layer impedance gradient to balance the usually conflicting goals of high absorption and broad bandwidth, hitting an EAB of 3.29–18 GHz and a minimum *RL* of −56.08 dB at the same time. As shown in Figure 12c, Zhao et al. [136] designed an ultra-wideband absorber for oblique-incidence service conditions, optimizing the absorption at normal incidence and at ±60° oblique incidence together.

Most current work covers two-objective optimization only (absorption plus weight or thickness). Pareto fronts in three or more dimensions remain under-explored, mainly because each extra objective dimension makes the sample size required to cover the front grow exponentially, and electromagnetic/mechanical/thermal multi-physics coupling further complicates the surrogate modeling.

For multi-objective optimization, the main challenge is not only finding a Pareto front but also determining whether the front is reliable and useful for engineering design. A Pareto-optimal point predicted by an ML model may be sensitive to measurement noise, fabrication tolerance, or small changes in thickness and filler content. Therefore, absorber optimization should report not only *RL* and EAB but also matching thickness, areal density, mechanical stability, thermal stability, cost, and processing feasibility when possible. Robust Pareto optimization, in which each candidate is evaluated under perturbations of thickness, composition, and electromagnetic parameters, is more meaningful than single-point optimization. Experimental confirmation of several representative Pareto candidates is also recommended, rather than validating only the predicted best point.

### 4.8. Data Augmentation and Few-Shot Learning

Data augmentation and few-shot learning tackle the data-scarcity bottleneck that runs through AI in absorbing materials—most experimental datasets contain only tens to hundreds of samples, and even simulation datasets usually sit in the hundreds-to-thousands range [38,137,138,139,140,141]. The paradigm squeezes model performance out of a limited data budget by generating virtual samples, transferring knowledge across tasks, or actively choosing the most informative training points [57,142,143].

The Chimeric VAE-LEFormer of Dong et al. [57] shows strong data efficiency: with only 2585 training samples, it realizes high-quality inverse generation of free-form metasurface absorbers across 8–18 GHz, thanks to its pretraining-plus-fine-tuning strategy—the pretrained VAE has already learned generic priors such as connectivity, symmetry and minimum feature size from a large set of unlabeled patterns, which cuts the target data needed for conditional generation. Ding et al. [142] studied how to shrink a dataset to accelerate metasurface inverse design, removing redundant samples through clustering and diversity sampling and training on the smallest distribution-covering subset. Wang et al. [143] proposed an active-sampling strategy that lets the model’s own predictive uncertainty guide the next sampling point and simulates or measures preferentially in the most uncertain region of parameter space, so that the same simulation or experimental budget produces the most informative dataset.

Cross-system transfer learning (e.g., transferring a model trained on carbon-based composites to MXene or high-entropy systems) is the likely next growth point of this paradigm; a unified foundation model for absorbing materials is the long-term target [120,144,145,146,147,148].

Data augmentation and few-shot learning can improve model training under data scarcity, but they cannot replace physically meaningful data. Synthetic samples generated by interpolation, generative models, or simulation may reproduce the statistical distribution of the original dataset while missing hidden experimental biases or processing uncertainties. Transfer learning can reduce the data requirement for a new material system, but negative transfer may occur when the source and target domains differ in loss mechanism, morphology, frequency range, or fabrication route. Therefore, augmented or transferred models should be validated on independent experimental samples from the target domain. Active learning is especially valuable because it does not merely enlarge the dataset, but selects the most informative new samples for simulation or experiment, making the data-generation process more efficient and physically grounded.

**Table 4:** Summary of the Eight Application Paradigms.

Paradigm	Core Methods	Methodological Note	Representative Works
4.1 Forward prediction	RF, XGBoost, DNN, Ensemble	Simple and reliable; cannot actively propose new designs	Che et al. [7,41,56]
4.2 High-throughput screening	ML + GA/PSO/BO	Explores spaces beyond human reach; convergence bounded by surrogate accuracy	Zhou et al. [7,42,125]
4.3 Inverse generation	VAE, GAN, MDN, Tandem	Handles one-to-many mapping and creates novel topologies; few experimental verifications so far	Dong et al. [43,57,84,128]
4.4 Parameter inversion/decoupling	DNN + tensor theory, locking strategy	Closer to physical essentials; sensitive to forward-model accuracy	Liu et al. [81,130]
4.5 Physics-informed modeling	PINN, DPD-NN, ECM + ML	Data-efficient and physically trustworthy; implementation-heavy and transferability unclear	Cao et al. [59,86,131]
4.6 Explainability	SHAP, Eigen-CAM, PDP/ICE	Turns statistical association into physical insight; reveals association, not causality	Shi et al. [44,56,87,133]
4.7 Multi-objective optimization	MOGA, multi-task learning	Aligns with engineering needs; high-dimensional Pareto search costs a lot	Wang et al. [42,135,136]
4.8 Data augmentation	Pretrain + fine-tune, active learning	Addresses the data-scarcity bottleneck; cross-system transfer not yet validated	Dong et al. [57,142,143]

## 5. Future Perspectives

### 5.1. Data Infrastructure and a Foundation Model for Absorbing Materials

The lack of standardized and shared data is the most basic bottleneck for AI in absorbing materials today. Almost every study uses its own small dataset (a few tens to a few thousand samples) with its own format, and meaningful comparison, reuse or integration across studies is hardly possible. Models stay in the one-task-one-dataset-one-model regime and cannot accumulate knowledge across tasks. The community needs to build a unified multimodal database that follows the FAIR principles (Findable, Accessible, Interoperable, Reusable), with consistent description of composition (filler type, content, morphology), processing (method, temperature, time, pressure), structure (thickness, layer number, porosity), electromagnetic properties ($\varepsilon_{r}(f)$, $\mu_{r}(f)$, *RL*(f), EAB), test conditions, and characterization images (SEM/TEM); NLP and LLM tools can then extract composition–processing–property relations from the existing literature to scale up the data volume. Such a database enables training a foundation model for absorbing materials—pretrained on data that crosses material systems, performance targets and characterization modalities to obtain a generic material representation, and then lightly fine-tuned for each downstream task—moving the field from task-specific small models to a general methodology.

To improve the realism and reusability of AI-ready absorber datasets, future studies should report not only the best absorption performance but also the complete experimental, simulation, and metadata information required to trace how each data point was generated. Table 5 summarizes a practical data quality checklist for FAIR reuse of electromagnetic wave absorbing material datasets.

Following such a checklist can reduce hidden dataset bias, improve cross-study comparability, and make ML models more reliable when they are used for screening, inverse design, uncertainty-aware optimization, and experimental recommendation.

### 5.2. Diffusion Models and Uncertainty-Aware Inverse Design

Diffusion models have replaced GANs as the state-of-the-art paradigm for image and molecule generation. Their core idea—corrupting data with noise step by step and learning the reverse denoising process—fits the structure of electromagnetic inverse design, since inverse design itself recovers a structure from a target performance specification. Compared with VAE and GAN, diffusion models deliver higher generation quality with more stable training, and physical constraints such as Maxwell consistency can be injected step by step during the denoising. This direction is empty in absorbing materials and opens a 2–3-year research window. A parallel direction is uncertainty quantification (UQ): current ML models usually output only a single best-guess prediction without telling researchers how much it can be trusted, which makes it hard to tell a high-confidence optimum from a low-confidence guess. Equipping every prediction with a credible interval through Bayesian neural networks, deep ensembles or conformal prediction, and feeding processing tolerances and measurement noise into the design stage, can move AI-based absorber design from single-point optimization toward robust design with manufacturable yield guarantees.

For example, in the inverse design of a metasurface absorber, each intermediate structure generated during the reverse diffusion process can be evaluated by a differentiable electromagnetic surrogate or full-wave solver. A Maxwell-residual or absorption-spectrum mismatch penalty can then be calculated and used as a gradient-guidance term to steer the next denoising step toward physically consistent structures. Alternatively, each generated pattern can be projected onto a physically admissible design space, such as satisfying minimum feature size, binary material distribution, passivity, and manufacturability constraints. In this way, diffusion-based generation is guided not only by learned data priors but also by electromagnetic physical consistency.

### 5.3. Cross-System Transfer Learning and Manufacturability Constraints

Cross-system transfer learning aims to break the strongly local nature of current AI applications, where a model trained on carbon-based composites cannot be reused for MXenes and one trained on metasurfaces cannot be reused for magnetic materials. Through fine-tuning or domain adaptation, models pretrained on data-rich systems can be transferred to data-scarce target systems such as high-entropy alloys, MXenes, and MOF-derived materials, offering a pragmatic path for the long-tail data scarcity in this field. An equally critical engineering bottleneck is the manufacturability constraint and engineering scale-up: most AI-designed absorbing structures and metasurface patterns to date exist only at the simulation level. Moving from a perfect digital design to a reproducible laboratory or production-line sample requires manufacturability constraints (minimum feature size, layer-to-layer alignment accuracy, processing temperature window, etc.) to be encoded explicitly in the AI design pipeline, cost, environmental impact and long-term durability to enter the objective function, and process-consistency strategies to be developed for batch fabrication. These steps move AI-designed materials from simulation-level optima to economically feasible and long-term, reliable products.

### 5.4. AI-Agent-Driven Self-Driving Laboratories

AI-Agent-driven self-driving laboratories represent an important future opportunity, but they remain largely undeveloped for electromagnetic wave absorbing materials. A full research workflow contains eight steps—literature review, hypothesis generation, structural design, electromagnetic simulation, sample fabrication, performance testing, data analysis, and hypothesis update—but AI today only participates in the simulation surrogate and design optimization steps. In the broader materials field, the A-Lab at Berkeley (2023) [5] closed a full loop in which AI generates hypotheses, robots carry out powder mixing and sintering, XRD performs the characterization, and AI then analyzes the results and designs the next experiment, synthesizing 41 novel inorganic solid-state materials without human intervention; platforms such as ARES show similar potential. Building such a loop for absorbing materials needs five cooperating components: a design Agent based on diffusion models or VAEs that proposes candidate structures and compositions, a simulation Agent that calls the CST/HFSS/COMSOL APIs for electromagnetic verification, an experiment Agent that drives the robotic platform for filler/matrix mixing, curing, and automatic VNA testing, an analysis Agent that uses interpretability tools such as SHAP and Eigen-CAM to extract physical insights and update the design strategy, and a knowledge Agent (built on an LLM) that retrieves the relevant literature and links new findings with prior knowledge—all working around a shared knowledge base. The physics-informed reinforcement learning work of Dong et al. [86] serves as an early prototype of the decision engine inside such a loop. The main open challenges include the strongly coupled multi-step processing of absorber samples (far more complex than the solid-state powder synthesis handled by A-Lab), the sim-to-real gap between idealized simulations and non-ideal experimental conditions, and the safety and reliability of autonomous decisions made by the Agents.

### 5.5. LLM-Assisted Design, Digital Twins, and Multiscale Modeling

The potential value of large language models (LLMs) in absorbing materials lies in three areas. A domain-fine-tuned LLM can extract composition–processing–property relations from tens of thousands of existing papers and build a knowledge graph for absorbing materials. Integrated with the Python (version 3.12) APIs of CST, HFSS, or COMSOL, an LLM can drive the simulation workflow through natural-language instructions and parse the results back into natural language. Paired with the generative inverse models discussed in Section 4, an LLM can act as an interactive design assistant that turns a vague design intent into a precise numerical objective and lays out multi-option trade-off analyses. The main risk is hallucination—generating material suggestions that look plausible but are physically wrong—so a mandatory physical verification step is essential. Digital twins build a high-fidelity virtual counterpart of a physical object (e.g., an absorbing coating on aircraft skin) and, by combining sensor data with ML degradation models, predict the remaining useful life in real time and, when paired with tunable metasurfaces, enable adaptive absorption during service. Multiscale AI modeling targets a longer-term goal: graph neural networks predict intrinsic $\varepsilon_{r}$ and $\mu_{r}$ from crystal structures at the first-principles level; CNNs or Transformers predict effective electromagnetic parameters from microscopic morphology at the mesoscale; physics-informed models integrate the predictions into full-wave simulation at the macroscale; and the three scales are bridged through a multi-fidelity framework. The end goal is an end-to-end pipeline that takes a crystal structure as input and outputs a macroscopic *RL* curve, a long-term target of computational electromagnetic materials science.

## 6. Conclusions

Artificial intelligence and machine learning are reshaping the research paradigm of electromagnetic wave absorbing materials. Taking the AI methodology as the main viewpoint, this review first laid out in Section 2 the three core physical quantities of absorbers—*RL*, Z, and α—and the performance ceiling set by the Rozanov and Snoek limits, and showed that AI-based optimization searches for a Pareto balance between impedance matching and high attenuation, while generative AI offers a way to approach the ceiling through topology innovation. Section 3 organized the field by model type and grouped existing or potentially valuable AI/ML methods into seven categories—classical machine learning, deep learning, generative models, large language models, AI Agents and self-driving labs, physics-informed and hybrid models, and optimization algorithms—together with a detailed table of representative works covering algorithms, material systems, and reported performance. Section 4 then organized the field by application paradigm and summarized the main practices in absorbing materials into eight paradigms—forward prediction, high-throughput screening, inverse generation, parameter inversion and decoupling, physics-informed modeling, explainability and mechanism mining, multi-objective and multifunctional co-optimization, and data augmentation and few-shot learning—moving from passive prediction, through active creation, to mechanism understanding. Section 5 laid out the key directions for the next 5–10 years: a unified FAIR-compliant multimodal database and a foundation model for absorbing materials, diffusion-based and uncertainty-aware inverse design, cross-system transfer learning together with manufacturability constraints, AI-Agent-driven self-driving laboratories, and frontier paradigms, including LLM-assisted design, digital twins, and multiscale modeling. AI has grown from an optional auxiliary tool into a core methodology for absorbing-material research. The goal is human–AI collaboration: researchers raise scientific questions and make value judgments; AI Agents handle the design, simulation, fabrication, testing and analysis tasks.

## Figures and Tables

**Table 1:** Workflow Taxonomy for ML-Assisted Absorber Design.

Aspect	Materials-Centric Absorbers	Metasurface/Structure-Based Absorbers
Design object	composition, processing, microstructure	geometry, topology, unit-cell pattern
Typical descriptors	filler type, loading, thickness, *εr*, *μr*, morphology	patterned images, periodicity, S_11_/S_21_, sheet resistance
Data source	VNA, literature data, synthesis/characterization	full-wave simulation, lithography/fabrication data
Main tasks	property prediction, composition screening, parameter decoupling	spectrum matching, topology generation, inverse structure design
Validation	RL, EAB, thickness, density, experimental test	S-parameters, absorptance, angular/polarization stability

**Table 2:** Representative AI/ML works in the electromagnetic wave absorbing material field.

Model/System	Year	Author	Model Type	Key Methodological Highlight	Application	Material System	Performance
MLFS	2023	R. Che [7]	RF + pattern recognition	High-throughput screening + inverse projection	Screening & forward prediction	Carbonyl iron/Fe_3_O_4_	*RL* increased by 207%, BW broadened by 360%
DCPRO	2025	R. Zhang [41]	Random Forest	Permittivity prediction + two-layer impedance gradient	Forward + impedance design	Flexible graphene composite	EAB 3.29–18 GHz, *RL_min_* −56.08 dB
Multi-ML Ensemble	2026	P. Jain [79]	XGB/CatBoost/RF/ET/KNN ensemble	Five-model parallel + weighted ensemble	Forward + inverse design	Multilayer absorbing metamaterial	R^2^ > 0.99
Weighted Ensemble + SHAP	2022	M. Shi [56]	RF/XGB/SVR/… weighted ensemble	Ensemble + SHAP + PDP/ICE rule mining	Forward + interpretation	Carbon/polymer nanocomposite	High-accuracy EMI SE prediction
Bayesian Active Learning	2024	J. Liu [80]	GPR + BO active sampling	Hundreds of simulations for broadband design	Active learning	ITO broadband metasurface	Lightweight + polarization-insensitive
NN Permittivity Engineering	2026	C. Liu [81]	DNN + tensor EM	Permeability-locking + permittivity-optimization	Decoupling + inverse	Flake carbonyl iron/BaTiO_3_	EAB 5.1 GHz @1.0 mm, *RL* −45.12 dB
MXene five-layer film ML design	2025	H. Zhou [82]	DNN	Sheet/hollow-sphere alternating structure	Structural design	MXene film	*RL*_min_ −48.15 dB, EAB 5.84 GHz
Lightweight CNN Graphene	2023	N. Chen [83]	CNN + transposed conv.	Tunable graphene conductivity as extra DOF	Forward + inverse	Graphene microwave metasurface	Multi-spectrum customizable absorption
LAM-LSTM	2025	H. Sun [84]	LSTM + local attention	Target S_11_ network + geometry recovery	Inverse design	Hexagonal honeycomb metasurface	8–17 GHz, MSE < 1.6 × 10^−4^
Chimeric VAE-LEFormer	2026	L. Dong [57]	VAE + Transformer	Dual VAE shared decoder + physics filter	Inverse generation	Free-form metasurface	8–18 GHz, 99% pattern fidelity
Explainable Adversarial Net	2024	N. Narang [85]	GAN + XAI	Explainability constraints in generation	Inverse + explanation	EMI shielding	Explainable generation
MDN Transparent Absorber	2025	X. He [43]	MDN	Multi-solution output + simulation check	Inverse design	Ionic-liquid transparent absorber	4.18–34.9 GHz, T 76.5%
Physics-Informed *RL*	2025	G. Dong [86]	*RL* + physics	*RL* decision + physics consistency	Optimization & design	Metasurface	Agent decision prototype
RF + PSO Multilayer Lattice	2025	L. Zhou [42]	RF + PSO	Volume-fraction gradient optimization	Structural optimization	Multilayer Octet lattice	Nonlinear gradient outperforms uniform/linear
DCNN + Eigen-CAM	2023	M. Shi [44]	ResNet + Eigen-CAM	Visualizing pore contribution from SEM images	Explainable mining	CNTs/PVDF porous composite	SE 105 dB @30 wt%

**Table 3:** Model-selection and validation guidance for AI-assisted electromagnetic wave absorbing materials.

Data Type/Task	Suitable Models	Typical Input Descriptors	Typical Outputs	Recommended Validation
Tabular composition–property prediction	RF, XGBoost, SVR, GPR, shallow DNN	Composition, filler loading, processing parameters, thickness, density, frequency, εr, μr	*RL*, EAB, $\varepsilon_{r}$, $\mu_{r}$, SE	R^2^, MAE, RMSE, sample-level train/test split, external test set
Spectral sequence prediction	LSTM, GRU, 1D-CNN, Transformer	Frequency-dependent $\varepsilon_{r}$(f), $\mu_{r}$(f), S-parameters, *RL* curves	Full *RL*(f), S_11_(f), absorption spectra	Curve-level MAE/RMSE, spectral-shape similarity, validation on unseen samples
SEM/TEM image analysis	CNN, ResNet, Vision Transformer, CNN + CAM	SEM/TEM images, morphology labels, porosity, filler distribution	SE, *RL*, morphology–property correlation	Image-level test split, classification accuracy, MAE/RMSE, CAM-based physical interpretation
Metasurface topology generation	CNN, VAE, CVAE, GAN, diffusion model, tandem network	Binary patterns, unit-cell geometry, periodicity, sheet resistance, target spectra	Candidate topology, S_11_/S_21_, absorptance	Forward-model verification, full-wave simulation, manufacturability check
Inverse design of composition or structure	VAE, CVAE, MDN, tandem network, GA/PSO + surrogate	Target *RL*/EAB, target frequency band, thickness constraint, material/structure variables	Multiple feasible compositions or structures	Forward prediction, physical filtering, full-wave simulation, experimental confirmation
Uncertainty-aware optimization	GPR, Bayesian optimization, deep ensemble, Bayesian neural network	Candidate design variables, surrogate prediction, uncertainty estimates	Optimized candidates, confidence interval, Pareto front	Uncertainty interval, acquisition-function trace, validation of top-ranked candidates
Physics-constrained modeling	PINN, physics-regularized DNN, ECM + ML, multi-fidelity model	$\varepsilon_{r}$, $\mu_{r}$, thickness, frequency, boundary conditions, physical equations	Physically consistent *RL*, S-parameters, field response	Physics-residual error, comparison with baseline ML, out-of-distribution test, experimental or simulation validation
Multi-objective design	Multi-task DNN, MOGA, PSO, BO, Pareto learning	Absorption, thickness, density, mechanical/thermal parameters, cost	Pareto-optimal candidates	Pareto-front stability, robustness under perturbation, experimental validation of representative candidates

**Table 5:** Data quality checklist for FAIR reuse of electromagnetic wave absorbing material datasets.

Category	Minimum Information to Report	Main Uncertainty or Inconsistency Controlled	FAIR Reuse Value
Material identity and composition	Material name, filler/matrix type, chemical composition, filler loading, element ratio, additive or dopant content, batch information if available	Ambiguous material naming, hidden composition differences, difficulty in comparing nominally similar samples	Enables searchable and machine-readable material identification
Processing and fabrication conditions	Synthesis route, mixing method, temperature, time, pressure, atmosphere, curing/sintering/carbonization conditions, post-treatment, coating or printing method	Processing-dependent microstructure variation, batch-to-batch fluctuation, fabrication tolerance	Supports reproducibility and process–structure–property analysis
Microstructure and morphology	SEM/TEM images, particle size, pore size, interface structure, layer stacking, filler dispersion, porosity, anisotropy, crystallinity or phase information	Hidden morphology differences, publication bias toward selected images, difficulty in linking microstructure to absorption mechanisms	Enables multimodal learning and image–property correlation
Sample geometry and normalization	Sample shape, length/width/diameter, thickness, matching thickness, areal density, bulk density, filling ratio, backing condition, number of layers	Thickness-dependent *RL*/EAB comparison, density-related performance overestimation, inconsistent lightweight evaluation	Allows fair comparison of absorption performance under equivalent geometry and weight
Electromagnetic measurement setup	VNA model, calibration method, coaxial/waveguide/free-space fixture, sample holder, frequency range, temperature, incident angle, polarization, repeated measurements if available	VNA calibration error, fixture mismatch, air gap, sample-holder effect, angular or polarization inconsistency	Improves traceability and cross-laboratory comparability
Electromagnetic parameter extraction	Raw S-parameters, extraction method such as NRW or free-space retrieval, assumptions used, smoothing or correction method, treatment of phase ambiguity, uncertainty of εr and μr	NRW extraction error, phase ambiguity, non-physical εr/μr, invalid homogeneity or isotropy assumptions	Allows recalculation, reanalysis, and consistency checking
Absorption-performance metrics	Full *RL*(f) curves, *RL* calculation equation, EAB definition, threshold used, frequency band, minimum *RL*, matching thickness, bandwidth-to-thickness ratio if used	Different *RL*/EAB definitions, overemphasis on *RL_min_*, missing frequency-window information	Enables standardized model training and fair performance ranking
Simulation data and model settings	Simulation software (Python 3.12), geometry file, boundary conditions, mesh size, material parameters, port settings, incident angle, polarization, convergence criteria	Simulation–experiment gap, idealized boundary assumptions, non-reproducible full-wave simulation results	Supports reproducible simulation datasets and multi-fidelity modeling
Validation protocol	Train/validation/test split strategy, sample-level or frequency-point-level split, external test set, cross-validation setting, experimental confirmation of predicted candidates	Data leakage, overestimated model accuracy, poor extrapolation to new material systems	Improves model trustworthiness and benchmark comparability
Uncertainty and error reporting	Measurement error, standard deviation from repeated tests, uncertainty intervals of $\varepsilon_{r}$, $\mu_{r}$, *RL* and EAB, model prediction uncertainty, fabrication tolerance analysis	Hidden noise, overconfident ML prediction, unreliable Pareto optimum or inverse design result	Supports uncertainty-aware optimization and robust design
Data completeness and bias control	Number of samples, number of failed or low-performance samples, negative results if available, selection criteria, missing-value treatment, literature-extraction rules	Publication bias toward best-performing samples, survivorship bias, incomplete metadata	Reduces dataset bias and improves generalization
Data accessibility and provenance	Raw data files, processed data, metadata schema, units, code or scripts, DOI or repository link, license, citation information, version number	Non-reusable datasets, unclear data provenance, unit inconsistency, loss of traceability	

## Data Availability

No new data were created or analyzed in this study.
